# Atrial cardiomyopathy: from knowledge gaps to clinical impact: The role of the EmbRACE Network

**DOI:** 10.1007/s12471-026-02081-3

**Published:** 2026-10-15

**Authors:** Nick L. van Vreeswijk, Zarina Habibi, Sander van Doorn, Joris R. de Groot, Monika Stoll, Daniël A. Pijnappels, Jordi Heijman, Robert G. Tieleman, Ron Pisters, Martin E. W. Hemels, Fleur V. Y. Tjong, Ulrich Schotten, Michiel Rienstra, Geert-Jan Geersing, Jelle C. L. Himmelreich

**Affiliations:** 1 Department of Cardiology, University of Groningen, University Medical Centre Groningen, Groningen, The Netherlands; 2 Department of Physiology, Cardiovascular Research Institute Maastricht (CARIM), Maastricht University, Maastricht, The Netherlands; 3 Department of Cardiology, Maastricht UMC+, Maastricht, The Netherlands; 4 Julius Centre for Health Sciences and Primary Care, University Medical Centre Utrecht, Utrecht University, Utrecht, The Netherlands; 5 Heart Centre, Department of Clinical and Experimental Cardiology, Amsterdam University Medical Centres, University of Amsterdam, Amsterdam, The Netherlands; 6 Department of Biochemistry, Maastricht University, Maastricht, The Netherlands; 7 Department of Genetic Epidemiology, Institute of Human Genetics, University of Münster, Münster, Germany; 8 Laboratory of Experimental Cardiology, Department of Cardiology, Heart Lung Centre Leiden, Leiden University Medical Centre, Leiden, The Netherlands; 9 Gottfried Schatz Research Center, Division of Medical Physics and Biophysics, Medical University of Graz, Graz, Austria; 10 Department of Cardiology, Martini Hospital, Groningen, The Netherlands; 11 Department of Cardiology, Rijnstate Hospital, Arnhem, The Netherlands; 12 Department of Cardiology, Radboud University Medical Centre, Radboud University, Nijmegen, The Netherlands; 13 Amsterdam Cardiovascular Sciences, Heart Failure and Arrhythmias, Amsterdam UMC, Amsterdam, The Netherlands; 14 Department of General Practice, Amsterdam UMC, University of Amsterdam, Amsterdam, The Netherlands; 15 Amsterdam Public Health, Personalized Medicine, Amsterdam, The Netherlands

**Keywords:** Atrial Fibrillation, Cardiomyopathies, Heart Failure, Stroke, Catheter Ablation, Artificial Intelligence, Biomarkers, Translational Medical Research

## Abstract

**Background:**

Atrial cardiomyopathy (AtCM) is increasingly recognised as an underlying atrial substrate contributing to atrial fibrillation (AF), heart failure (HF), and cardio-embolic stroke. However, diagnostic criteria, disease thresholds, and targeted treatment strategies remain poorly defined.

**Objective:**

To describe the rationale, structure, and translational strategy of the EmbRACE (Electro-Molecular Basis and the theRapeutic management of Atrial Cardiomyopathy, fibrillation and associated outcomEs) network, a national consortium established to address key knowledge gaps in AtCM.

**Methods:**

EmbRACE integrates mechanistic research, computational modelling, clinical trials, and implementation science within seven interconnected work packages. Human atrial tissue biobanking and deep phenotyping (WP1) enable molecular profiling of candidate pathways (WP2), which are validated in experimental models (WP3). Computational modelling and artificial intelligence (WP4) integrate multimodal clinical and biological data to derive composite AtCM phenotypes and severity frameworks. Substrate-targeting clinical trials evaluate AF ablation (RACE X; WP5), weight reduction using GLP‑1 agonists (DUTCH-WAIST; WP6), and optimised rate control using calcium channel antagonists (SMART-VERAPAF; WP6). Implementation science (WP7) focuses on guideline integration, decision-support tools, and governance of AI-based risk models.

EmbRACE establishes a translational pipeline from mechanistic discovery to clinical application. By integrating tissue-level insights with multimodal risk modelling and interventional trials, the consortium aims to define clinically meaningful AtCM thresholds, to validate indirect markers, and to determine whether targeted treatment modifies disease progression and clinical outcomes.

**Conclusion:**

EmbRACE provides a multidisciplinary framework to transform AtCM from a theoretical construct into a clinically actionable entity, enabling earlier detection, improved risk stratification, and AtCM-directed therapy in cardiovascular prevention.

## What’s new?

- EmbRACE is a nationwide Dutch consortium dedicated to atrial cardiomyopathy (AtCM), integrating mechanistic research, artificial intelligence, clinical trials, and implementation science within one translational framework.
- The network moves beyond AF by targeting underlying AtCM across its molecular, structural, and functional dimensions.
- Computational modelling and AI are used to derive composite AtCM phenotypes and severity frameworks from multimodal data.
- Three substrate-oriented clinical trials (RACE X, DUTCH-WAIST, SMART-VERAPAF) evaluate whether targeting AtCM modifies disease progression and outcomes.
- EmbRACE provides a blueprint for interdisciplinary collaboration and national cardiovascular prevention strategies focused on atrial disease.

## Introduction: what is atrial cardiomyopathy, and what remains unknown?

Atrial cardiomyopathy (AtCM) is broadly defined as *“any complex of structural, architectural, contractile, or electrophysiological changes affecting the atria with the potential to produce clinically relevant manifestations”* [1]. The consensus definition underscores that AtCM encompasses diverse atrial remodelling processes, fibrosis, myocyte dysfunction, and conduction abnormalities that form the substrate for atrial arrhythmias and other clinical sequelae. In particular, AtCM is now recognized as an underlying substrate that contributes to atrial fibrillation (AF), heart failure (HF), and cardio-embolic stroke [2]. As the consequences of AtCM become more evident and treatment options are not well defined, there is a need to elucidate the underlying pathophysiological pathways and identify effective treatments.

**Fig. 1:**
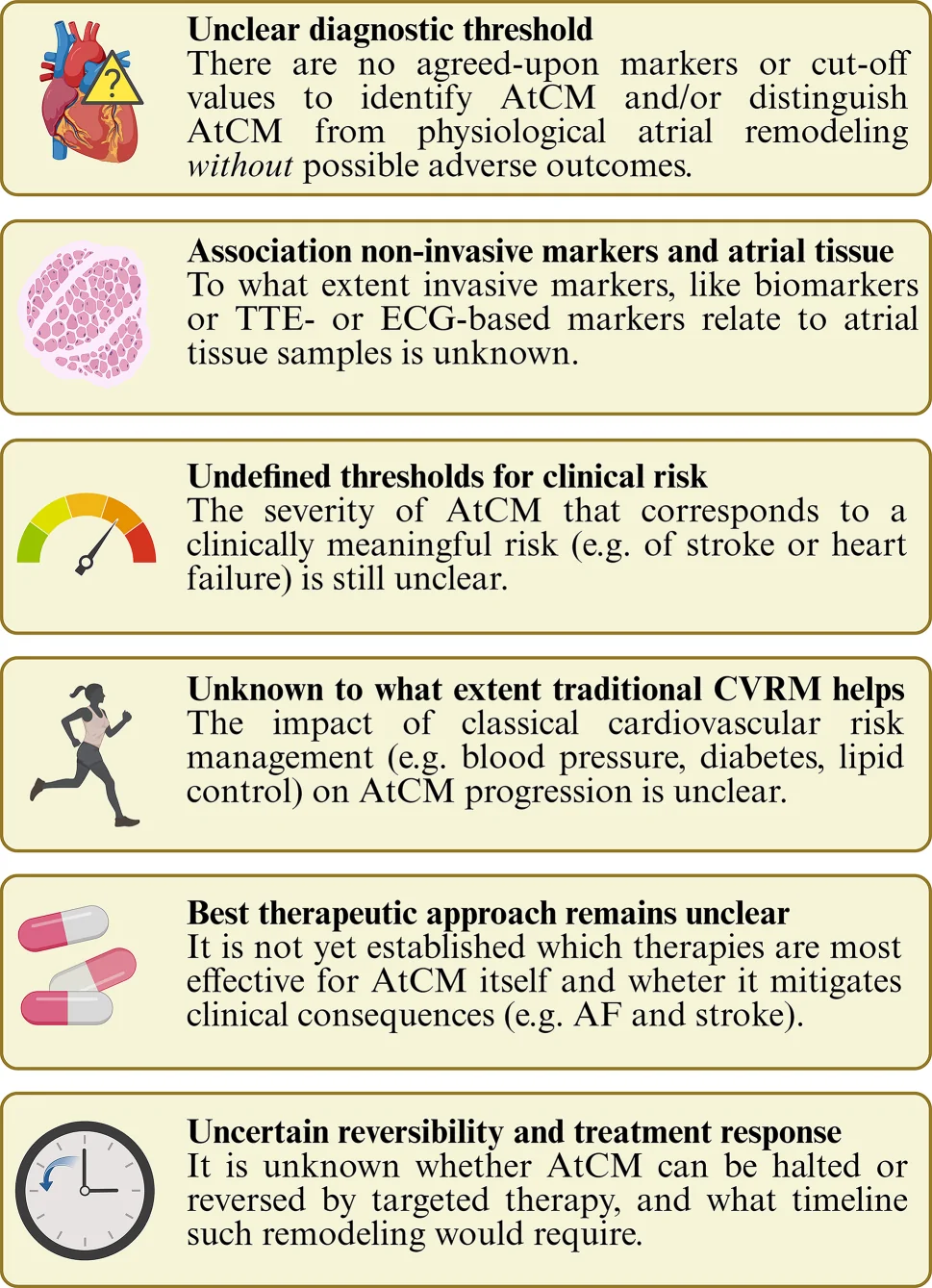
*Key knowledge gaps in atrial cardiomyopathy. *Summary of the major unresolved questions that currently hinder the translation of AtCM into clinical practice. Abbreviations: *AtCM* atrial cardiomyopathy, *CVRM* cardiovascular risk management, *ECG* electrocardiogram, *TTE* transthoracic echocardiogram

Many knowledge gaps on the prevention, identification, and effective treatment of AtCM remain (Fig. 1). The primary knowledge gap is the lack of agreed-upon markers or cut-offs for AtCM, with studies using heterogeneous imaging, electrocardiographic, histological, and biomarker criteria [1, 3, 4]. Additionally, the extent to which indirect markers of AtCM correlate with true atrial tissue architecture and composition is largely unknown. Similarly, it is unknown what threshold of AtCM results in a clinically significant risk of cardiac or extracardiac complications. Meanwhile, therapeutic approaches remain focused on the consequences of AtCM, such as AF, HF or stroke, rather than directly targeting AtCM itself. It is also unknown whether AtCM can be halted or reversed if appropriately addressed, and what time course such changes might require [5]. Moreover, whether treatment of AtCM mitigates clinical consequences of AtCM, such as AF, HF or stroke, remains unclear. Current, more traditional cardiovascular prevention frameworks pay little attention to atrial pathophysiology. Consequently, AtCM often progresses unnoticed until clinical consequences develop, making it a *pressing research priority* to bridge the knowledge gaps.

The EmbRACE network (*Electro-Molecular Basis and the theRapeutic management of Atrial Cardiomyopathy, fibrillation and associated outcomEs*) is a national, translational consortium preclinical, computational, and clinical research groups positioned to address the knowledge gaps relating to AtCM. By presenting the work packages (WPs) within EmbRACE, we demonstrate how the consortium integrates mechanistic research (WP1–3), clinical trials (WP5–6), and implementation (WP7) to address AtCM from basic science to academic cardiology and back. A key opportunity to bridge the gaps is the use of advanced computational modelling and artificial intelligence (AI; WP4). AI can integrate markers of AtCM across ECG, imaging, biomarkers, clinical profiles, and longitudinal trajectories to (i) derive composite AtCM phenotypes, (ii) quantify disease severity and progression, and (iii) support clinically meaningful thresholds for ‘test-and-treat’ strategies. Finally, we will provide future examples of how EmbRACE’s findings could be implemented in key clinical settings.

## Pathophysiological mechanisms underlying AtCM

A central mechanism in AtCM is progressive fibrosis [6]. Pressure and volume overload induce mechanical stretch, which triggers extracellular matrix deposition and structural remodelling [7]. Resulting collagen crosslinking disrupts fibre architecture, slows conduction, and promotes re-entry, and impairs atrial function [8].

The development and progression of fibrosis are amplified by multiple biological pathways. Oxidative stress contributes to cellular injury and dysregulation of fibroblast signalling [9]. Inflammation, characterised by cytokine activation and inflammasome signalling, further promotes fibrosis and electrical instability [10]. Metabolic dysregulation, including diabetes-related pathways, enhances oxidative stress, myocardial remodelling, and atrial dysfunction [11]. In addition, epicardial adipose tissue exerts local paracrine effects through pro-inflammatory and profibrotic mediators and may contribute to fibrofatty infiltration of the atrial myocardium [12].

## Frameworks for diagnosing and staging AtCM

Although a universally accepted definition of AtCM is lacking, several groups have proposed clinically applicable frameworks that integrate clinical and imaging markers and circulating biomarkers. Goette et al. classified AtCM into three stages, ranging from subclinical abnormalities without overt arrhythmia to ‘advanced atrial failure’ characterised by severely reduced atrial function, extensive fibrosis, atrial enlargement, and/or AF progression [1]. Similarly, Weerts et al. proposed a staged model that begins with electrical dysfunction (e.g. prolonged P‑wave duration or interatrial block), progresses to mechanical dysfunction (reduced atrial strain), atrial enlargement, and ultimately atrial failure characterised by persistent AF [13].

Importantly, evidence from large prospective cohorts integrating ECG, imaging, and circulating biomarkers has demonstrated that an increasing burden of AtCM markers is associated with a stepwise increase in cardiovascular events, including AF, HF, and stroke [2, 14].

## The EmbRACE network: a multidisciplinary strategy to address knowledge gaps in AtCM

**Fig. 2:**
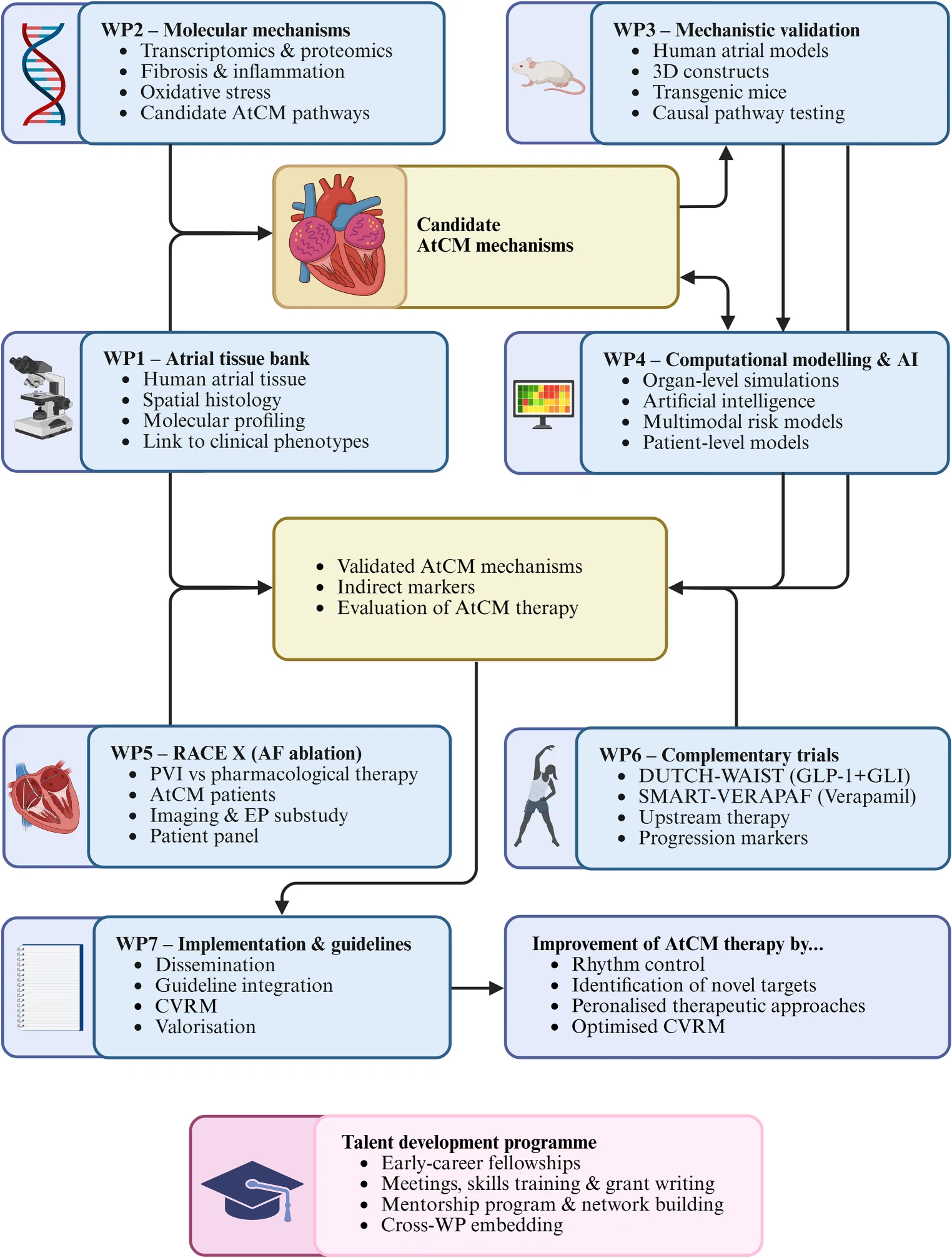
*Overview of the EmbRACE consortium and its translational research pipeline. *EmbRACE integrates human atrial tissue biobanking (WP1), molecular profiling (WP2), and experimental validation (WP3) to identify candidate AtCM mechanisms. These are integrated through computational modelling and AI-based risk prediction (WP4) and translated into substrate-targeting clinical trials (WP5–6). Validated mechanisms, indirect markers, and therapeutic strategies feed into implementation and guideline development (WP7). Abbreviations: *AF* atrial fibrillation, *AI* artificial intelligence, *AtCM* atrial cardiomyopathy, *DUTCH-WAIST* DUTCH Weight Control in Atrial Fibrillation Study, *GLP‑1* glucagon-like peptide‑1, *RACEX* AF ablation to prevent disease progression of AF-induced atrial cardiomyopathy in women and men, *SMART-VERAPAF* Self-management and random therapy with verapamil or metoprolol in paroxysmal atrial fibrillation, *WP* work package, *3D* three-dimensional

The EmbRACE network is a Dutch nationwide initiative founded in 2023 and funded by the Dutch Heart Foundation to improve understanding, diagnosis, risk stratification, and management of AtCM (Fig. 2). EmbRACE builds on *The Reappraisal of AF: Interaction Between Hypercoagulability, Electrical Remodelling, and Vascular Destabilization in the Progression of AF* (RACE V) study, which followed a cohort of 612 paroxysmal AF patients after implantation of a loop recorder and comprehensive deep phenotyping. Bringing together experts in electrophysiology, cardiology, molecular biology, computational modelling, bioinformatics, health economics, general practice, and clinical trial and implementation methodology, EmbRACE integrates mechanistic and computational insights with clinical validation and implementation. By incorporating sex-specific analyses across all work packages, EmbRACE aligns with national priorities to improve cardiovascular risk stratification and treatment. While founded as a consortium of cardiovascular researchers from Dutch institutions, the EmbRACE network welcomes international researchers and clinicians to participate throughout the initiative, forming a network of AtCM-interested investigators. EmbRACE is also designed as an open network that new researchers can join, with the ambition of promoting a sustainable, collaborative, and long-lasting research environment.

## EmbRACE work packages: research focus and clinical relevance

### Deep phenotyping and biobanking

Deep phenotyping of the atria at both the tissue and patient levels is a key pillar of EmbRACE (WP1). The network is establishing a high-quality biobank of human atrial tissue, collected during cardiac surgery and thoracoscopic AF ablations. The tissue bank builds on the RACE V tissue bank, integrating new cohorts. Well-characterized samples enable spatially resolved analyses of atrial remodelling on histological and molecular levels, and each donor patient will undergo detailed clinical phenotyping [15]. The tissue bank facilitates investigation of structural atrial changes and their histological characteristics, in patients with and without AF, albeit those without AF by definition had an indication to undergo cardiac surgery. Nevertheless, by linking atrial tissue to non-invasive clinical measurements (biomarkers, ECG, imaging), we aim to improve our interpretation of how these non-invasive tests may reveal about underlying tissue- and cellular-level pathology.

### Molecular mechanisms and experimental validation

Through advanced transcriptomic and proteomic profiling using the atrial tissue biobank of WP1, investigators aim to uncover pathophysiological pathways on the molecular and cellular pathways, such as fibrosis, inflammation, and oxidative stress in WP2 [16]. In WP3, *in vitro* and *in vivo* experimental models validate the mechanistic pathways of AtCM [17]. Engineered human atrial cell cultures, 3D tissue constructs, and genetically modified animal models enable the evaluation of causal relations between candidate pathways and AtCM (progression). Mechanistic studies investigate which molecular pathways are responsible for remodelling that accelerates AtCM [18]. Identified pathways may ultimately translate into novel biomarkers or therapeutic targets, which could, in the future, improve detection and treatment of AtCM [16].

### Computational modelling and risk prediction

EmbRACE employs computational modelling and AI to improve detection, staging, and risk prediction of AtCM (WP4). On the mechanistic side, organ-level simulations will integrate structural and electrophysiological data derived from tissue studies to investigate how AtCM promotes AF, producing in silico hypotheses for clinical validation [19]. On the data-driven side, AI models will be trained on multimodal clinical datasets (e.g., ECGs, Holter, echocardiographic and/CMR-derived atrial metrics, blood biomarkers, comorbidity profiles, and longitudinal outcomes) to identify subtle AtCM signatures that are not captured by single markers. Candidate approaches include representation learning to derive low-dimensional AtCM ‘health-space’ embeddings from ECG and imaging, as well as multimodal fusion models that integrate electrical, structural, and biochemical data. Finally, longitudinal patient-level models will be developed to quantify AtCM and AF progression and predict clinically relevant outcomes [20].

WP4 will emphasize interpretability and safety to support clinical adoption: uncertainty-aware predictions (calibration), clinically meaningful explanations, and external validation. Ultimately, WP4 aims to move from heterogeneous proxy markers to an integrated AtCM severity framework which can inform thresholds for downstream testing and intervention.

### Interventional trials targeting the substrate

EmbRACE is conducting three complementary clinical trials evaluating treatment strategies for AtCM. The first, *AF ablation to prevent disease progression of AF-induced atrial cardiomyopathy in women and men* (RACE X; WP5; NCT06200311), is a multicentre randomised trial investigating whether AF ablation can reduce AtCM-related outcomes. Notably, RACE X was co-designed with patients and incorporates patient-reported outcome measures to capture quality of life. RACE X includes patients irrespective of AF-related symptoms and therefore differs fundamentally from earlier ablation trials, which primarily enrolled symptomatic AF patients [21, 22].

As obesity and its comorbidities promote AtCM, weight loss has been suggested to target AtCM. The second trial, the *DUTCH weight control in AF study* (DUTCH WAIST; WP6; EU-CTR 2022-501392-81-00), therefore targets this modifiable risk factor that is often overlooked but is an important driver of AtCM: obesity. This placebo-controlled, double-blind study evaluates the prescription of the glucagon-like peptide (GLP‑1) receptor agonist (*Semaglutide*) on top of a structured lifestyle intervention to determine whether this approach is superior in delaying AF (progression).

The third trial, the *Self-management and random therapy with verapamil or metoprolol in paroxysmal AF *(SMART-VERAPAF; WP6; EU-CTR number 2025-524245-27), addresses outcomes of optimized rate control in paroxysmal AF. This randomised study compares verapamil versus metoprolol. The study features a centrally guided self-management approach using smartwatch-based monitoring to optimise treatment. Verapamil is a calcium-channel blocker which has been shown to reduce atrial electrical and contractile remodelling. Therefore, it is hypothesized that verapamil reduces AF progression, while improving rate control and patient well-being.

Importantly, all trials include sub-studies using non-invasive measurements such as echocardiography to assess how different interventions affect AtCM progression. If successful, these clinical trials could lead to new paradigms where targeting AtCM progression is a cornerstone of preventing AF, HF, and stroke.

### Implementation into clinical practice

To ensure that new knowledge truly benefits patients, a dedicated work package (WP7) focuses on clinical implementation and guideline development. Standardized diagnostic criteria for AtCM and practical tools to support clinical decision-making for physicians. By collaborating with professional societies and patient organizations, EmbRACE aims to facilitate the incorporation of AtCM into guidelines and educate health care professionals about *when and how to identify* on AtCM. WP7 will address governance requirements for AI-enabled tools, including pre-specification of intended use, dataset shift monitoring, periodic recalibration, and transparent reporting of performance across subgroups and care settings.

### Talent development programme

EmbRACE includes a cross-cutting *Talent Development Programme* to accelerate early-career researchers in AtCM through masterclasses, skills workshops, and mentorship and coaching. To attract and retain talent, EmbRACE funds three Early Career Fellowships (€ 50k; up to 12 months) and four Out-of-the-Box grants (€ 25k) to generate pilot data and support acquisition of external personal funding. Fellows are embedded within a work package with cross-consortium supervision, present their work at consortium meetings, undertake a visit where relevant, and are supported in a personal grant submission.

## Key settings for potential future clinical use of EmbRACE’s findings

**Fig. 3:**
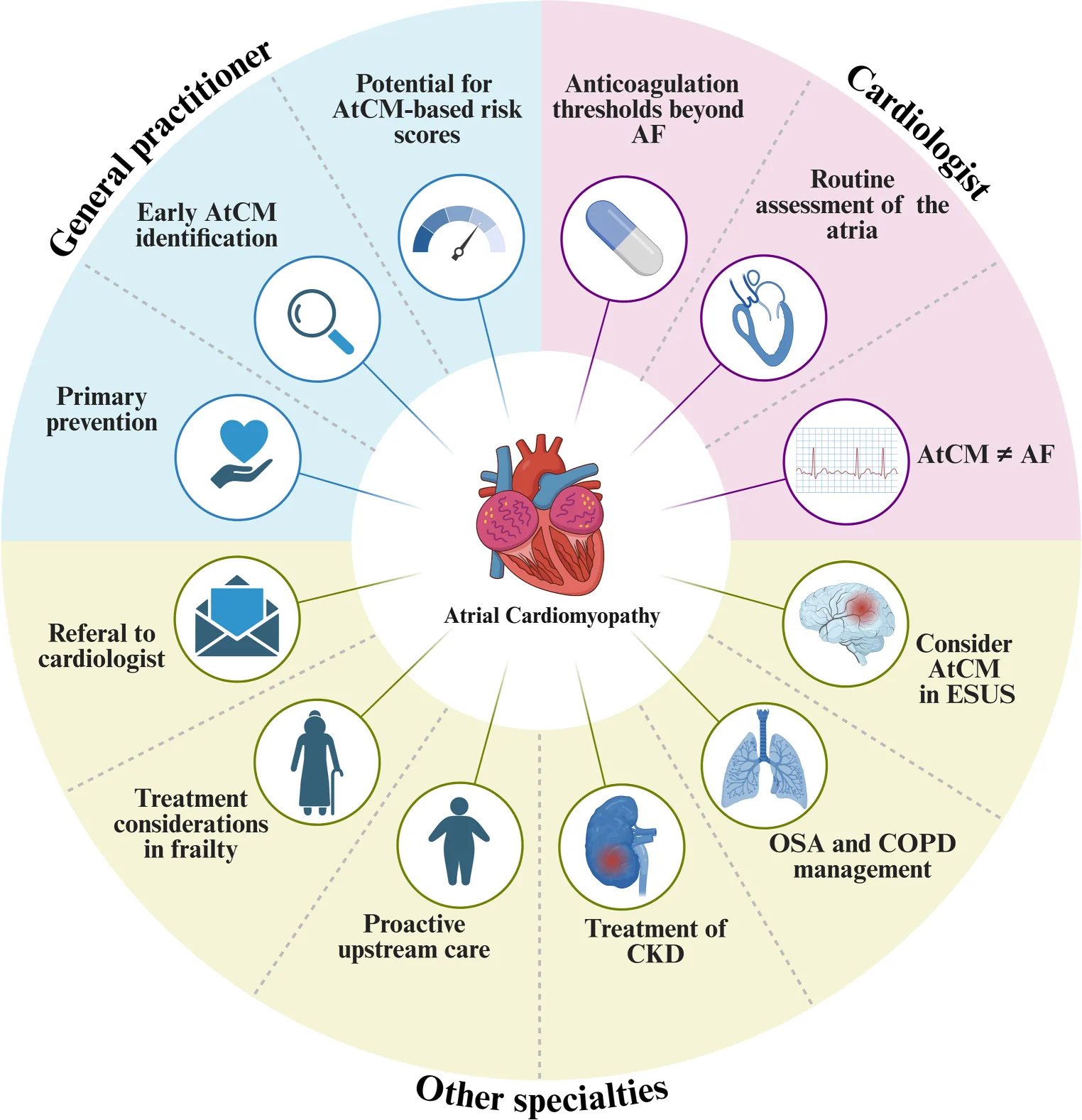
*Potential clinical implications of atrial cardiomyopathy for general practitioners, cardiologists, and other medical specialties. *This conceptual framework illustrates how emerging insights into AtCM may influence future clinical practice across the healthcare continuum. For general practitioners, early recognition of AtCM, integration of AtCM into CVRM, and improved referral patterns may enhance primary prevention and timely specialist involvement. For cardiologists, routine assessment of atrial structure and function, recognition that AtCM does not equal AF, and consideration of OAC based on AtCM rather than arrhythmia alone may support a more substrate-oriented approach to care. Among other specialties, including neurology, pulmonology, nephrology, and geriatrics, greater awareness of ACMP may improve evaluation of ESUS, guide management of comorbidities such as OSA, COPD, and CKD, and support frailty-adapted treatment strategies. Importantly, this figure reflects an early theoretical concept of how care *could* evolve if future research supports these pathways; many of the proposed applications remain hypothetical and require dedicated investigation before being implemented in routine clinical practice. Abbreviations: *AF* atrial fibrillation, *AtCM* atrial cardiomyopathy, *CKD* chronic kidney disease, *COPD* chronic obstructive pulmonary disease, *ESUS* embolic stroke of undetermined source

EmbRACE aims to ultimately empower clinicians to deliver tailored care, reduce unnecessary referrals, and prevent missed opportunities for upstream intervention. Below, we highlight a number of key settings in which lessons from EmbRACE may affect future clinical care (Fig. 3).

### Primary care: early AtCM detection and treatment; redefining AF

AtCM often develops silently and is driven by conditions commonly encountered and frequently first diagnosed in primary care, such as hypertension, diabetes, obesity, and sleep apnoea [1, 23]. Early detection of AtCM could offer an early opportunity to reduce the risk of AF, HF, and stroke. With its key role in both primary and secondary cardiovascular prevention as well as chronic disease management, primary care therefore may be well positioned to explore the use of AtCM in cardiovascular risk management. Current cardiovascular prevention programs largely focus on atherosclerotic disease, including lipid profiles, leaving potential for AtCM in improving cardiovascular risk stratification and management. AI-enabled triage may be relevant in primary care, where multimodal, accessible AtCM markers could be employed to guide referral, intensified risk-factor management, or rhythm surveillance. Although obviously there is overlap between current lipid-driven cardiovascular disease management, distinct differences could also be noted in a program more focussed on preventing AtCM (progression), such as i) more focus on sleep apnoea, ii) more focus on cardiometabolic risk via the relatively ‘novel’ disease modifying drugs like SGLT-2 inhibitors or incretin-based therapies, iii) more focus on early detection of HF, notably likely HFpEF, and iv) potentially in the future earlier strategies for stroke prevention with anticoagulants possibly even in the absence of a clinical AF diagnosis.

AF is regularly diagnosed and treated by GPs [24]. Although AF is widely recognized in Dutch primary care [25], the recognition of AtCM and its consequences are less established in primary care. An important advantage of AtCM over AF is that the detection of AF is hampered by its paroxysmal nature. Identifying patients with an overt underlying AtCM is therefore crucial to adequate risk stratification and appropriate intervention.

### Cardiology: recognising AtCM beyond rhythm disorders

As awareness of AtCM increases, the outputs of EmbRACE may encourage cardiologists to adopt a broader lens when interpreting ECGs, echocardiograms, or biomarker results, especially in patients who do not initially present with AF. More attention to left atrial size, reservoir strain, P‑wave indices, or circulating biomarkers could help cardiologists identify AtCM and enable intervention before clinical consequences occur. When AtCM is suspected or identified, cardiologists may also take a more proactive approach to managing upstream risk factors, in close collaboration with GPs and other involved physicians. With adequate recognition, AtCM could function as an actionable label to drive earlier and integrated intervention, even in patients referred for unrelated cardiovascular complaints.

### Interdisciplinary care: bridging heart, brain, and systemic comorbidity

Emerging evidence suggests that AtCM may contribute not only to stroke risk but also to cognitive dysfunction, even in the absence of clinical AF [26]. Neurologists may consider AtCM testing as part of the diagnostic work-up in the future. Recognising the role of AtCM in neurological dysfunction could encourage neurologists to employ more comprehensive cardiac phenotyping, including atrial imaging, biomarkers, or ECG parameters, even in patients without AF.

Given the interplay between AtCM and systemic comorbidity, internists and nephrologists may become increasingly attentive to AtCM in the context of chronic kidney disease, endothelial dysfunction, or metabolic dysregulation. As chronic pulmonary disease, including obstructive sleep apnoea and COPD, are associated with atrial stretch, inflammation, and autonomic dysregulation, there may be a role for pulmonary medicine in AtCM management. Identifying AtCM in patients with pulmonary disease may facilitate earlier referral to cardiology, optimisation of treatment, or proactive rhythm surveillance. Geriatricians may also play a key role. Frail older adults often present with multimorbidity, subtle cognitive decline, or non-specific symptoms such as fatigue or falls. In geriatric patients, AtCM could contribute to complex clinical syndromes that do not fit neatly traditional cardiovascular categories. Therefore, AtCM-focused assessment could ultimately support more holistic, frailty-adapted care strategies.

While still partly speculative, a broader interdisciplinary perspective reflects a growing need to integrate AtCM across organ systems. A holistic approach underscores that AtCM is not only an isolated cardiac condition, but that AtCM has bidirectional interactions spanning neurology, nephrology, pulmonology, geriatrics, and cardiovascular medicine.

## Conclusion

By systematically targeting key knowledge gaps, the EmbRACE network aims to develop an evidence-based, clinically relevant framework for AtCM. Uniting basic science, advanced imaging, computational modelling, and pragmatic clinical trials within a single collaborative framework, the network is positioned to transform AtCM from a theoretical concept into a clinically actionable entity. If successful, EmbRACE may enable earlier detection, more precise risk stratification, and more targeted treatment of AtCM, bringing it into the heart of cardiovascular prevention.

## Data Availability

All data supporting the findings of this work are included in the article.
